# Barriers to Malaria Control among Marginalized Tribal Communities: A Qualitative Study

**DOI:** 10.1371/journal.pone.0081966

**Published:** 2013-12-20

**Authors:** Radhika Sundararajan, Yogeshwar Kalkonde, Charuta Gokhale, P. Gregg Greenough, Abhay Bang

**Affiliations:** 1 Brigham and Women's Hospital, Boston, Massachusetts, United States of America; 2 Society for Education, Action and Research in Community Health (SEARCH), Gadchiroli, Maharashtra, India; University of California, San Francisco, United States of America

## Abstract

**Background:**

Malaria infection accounts for over one million deaths worldwide annually. India has the highest number of malaria deaths outside Africa, with half among Indian tribal communities. Our study sought to identify barriers to malaria control within tribal populations in malaria-endemic Gadchiroli district, Maharashtra.

**Methods and Findings:**

This qualitative study was conducted via focus groups and interviews with 84 participants, and included tribal villagers, traditional healers, community health workers (CHWs), medical officers, and district officials. Questions assessed knowledge about malaria, behavior during early stages of infection, and experiences with prevention among tribal villagers and traditional healers. CHWs, medical officers, and district officials were asked about barriers to treating and preventing malaria among tribal populations. Data were inductively analyzed and assembled into broader explanation linking barriers to geographical, cultural and social factors. Findings indicate lack of knowledge regarding malaria symptoms and transmission. Fever cases initially present to traditional healers or informal providers who have little knowledge of malaria or high-risk groups such as children and pregnant women. Tribal adherence with antimalarial medications is poor. Malaria prevention is inadequate, with low-density and inconsistent use of insecticide-treated nets (ITNs). Malaria educational materials are culturally inappropriate, relying on dominant language literacy. Remote villages and lack of transport complicate surveillance by CHWs. Costs of treating malaria outside the village are high.

**Conclusions:**

Geographic, cultural, and social factors create barriers to malaria control among tribal communities in India. Efforts to decrease malaria burden among these populations must consider such realities. Our results suggest improving community-level knowledge about malaria using culturally-appropriate health education materials; making traditional healers partners in malaria control; promoting within-village rapid diagnosis and treatment; increasing ITN distribution and promoting their use as potential strategies to decrease infection rates in these communities. These insights may be used to shape malaria control programs among marginalized populations.

## Introduction

Malaria infection is a major public health concern, thought to cause more than one million deaths in the world every year [1]. India has the highest number of malaria deaths outside of the African continent with an estimated 200,000 deaths annually [2]. Approximately 50% of all malaria deaths in India occur among members of tribal groups [3]. As tribal persons constitute less than 10% of India's total population, these communities bear a disproportionately heavy burden of disease.

Malaria control activities in India are carried out through the direction of the World Bank-funded National Vector Borne Disease Control Programme (NVBDCP). Strategies for malaria control employed by NVBDCP include: 1) early detection and prompt treatment of malaria cases 2) vector control using methods such as insecticide treated nets (ITN) and indoor residual spray (IRS) with Deltamethrin, 3) reducing breeding of mosquitoes by environmental management and source reduction and 4) community participation to control mosquito breeding [4]. While mortality and infection rates show declining trends in many regions of India as a result of this program [5], tribal regions of India continue to have high prevalence and mortality due to malaria.

In order to address the heavy burden of malaria in tribal regions, the NVBDCP has developed a vulnerable communities' plan (VCP), acknowledging that service delivery, vector management and community mobilization needs to be improved in these regions [6]. The action plan proposed under the VCP stresses early case detection and management, and arranging referrals to healthcare providers to avert morbidity and mortality. However, in tribal areas, health care seeking behaviors and healthcare delivery are significantly affected by socio-cultural and geographic factors. Understanding these factors can provide crucial insight towards improving malaria control strategies. This study seeks to understand the barriers to malaria control by exploring the factors that shape 1) healthcare seeking behavior among tribal populations and 2) healthcare delivery among tribal populations by NVBDCP workers in the malaria-endemic Gadchiroli district of India.

## Methods

### Study Site

**Figure 1:**
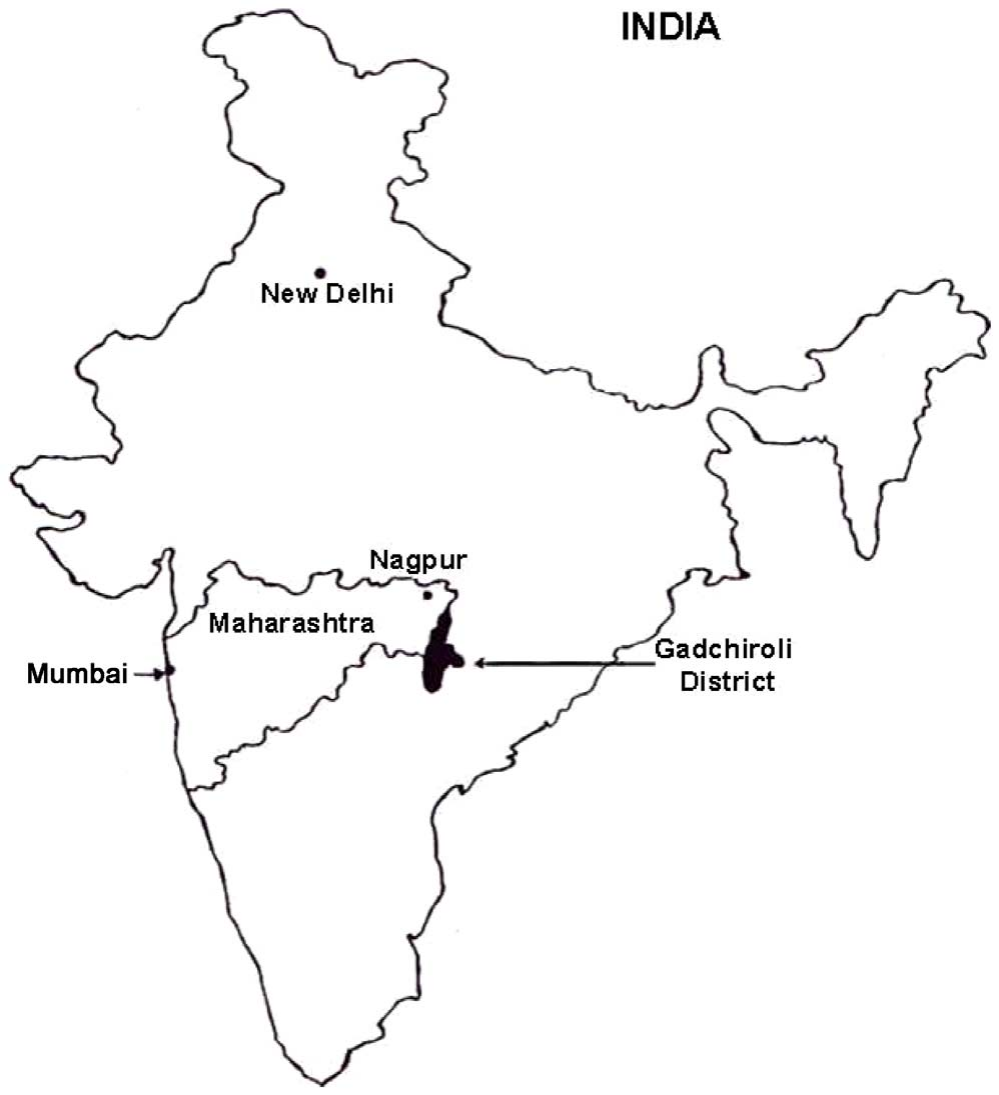
Gadchiroli District, Maharashtra State, India. Figure courtesy of SEARCH.

Gadchiroli district, along the southeastern border of the state of Maharashtra in central India (Figure 1), includes a population of approximately 1 million people, with a majority (93%) living in rural areas among 1679 villages [7]. About 75% of the 14,000 square kilometer geographical area of the district is covered by forest [7]. Over one-third of the district comprises members of the tribal groups – mainly Gond, Rajgond, Madia and Pardhan [8]. Tribal people in this district live in forested areas and earn their livelihood through farming and gathering forest produce such as bamboo, leaves, and medicinal herbs. Migration to urban areas for seeking jobs is minimal. The Government of India considers these populations *vulnerable communities*, or “… groups of people with social, cultural, economic, and/or political traditions and institutions distinct from the mainstream or dominant society that disadvantage them in the development process” [9].

Healthcare is provided in this district by government facilities, which include one district hospital, 12 rural hospitals, 45 primary health centers and 376 primary health units [7]. The NVBDCP is implemented in the district by District Malaria Officer (DMO), with physicians stationed at the Primary Health Centers (PHCs), and community health workers (CHWs) who travel to villages to provide testing, treatment, and distribution of malaria chemoprophylaxis. Rapid diagnostic tests are available in some areas, though in most regions malaria testing is done solely with blood smear slide microscopy.

Gadchiroli district has persistently high rates of malaria infection, and is considered a chloroquine resistant region [10]. NVBDCP data reports 11,694 confirmed cases of malaria in year 2011–12. Over the last five years, Gadchiroli district had 61,399 malaria cases, which accounted for 12% of total malaria cases in the state of Maharashtra [11]. This is a disproportionate burden as the population of Gadchiroli district accounts for only 1% of the state's total population. The slide positivity rate for this district in last five years has remained between 1.4 and 2.5 despite malaria control efforts, and was 2.2 in 2011–2012 [11]. Approximately 80% of malaria cases in this district are caused by *Plasmodium falciparum* [11]. Malaria transmission in the region generally occurs between July and December, with the peak in November.

The Society for Education, Action and Research in Community Health (SEARCH) is a non-governmental organization working in Gadchiroli district for the last 25 years. SEARCH has established a community healthcare network among tribal communities in this region, which includes 42 villages with a population of approximately 10,000 people. Village health workers (VHWs) employed by SEARCH provide healthcare for selected diseases, including malaria, in these villages.

In order to understand barriers to malaria control in this region, it is first imperative to consider the geographic and socio-cultural contexts of tribal life.

#### Geographic factors

Tribal communities in modern India tend to inhabit remote, often heavily forested, and rural areas. A 1994 survey by the Indian Ministry of Rural Development found that half of rural tribal populations were below the national poverty line [12]. Many tribal people do not have formal land rights. Further, the geographic isolation of Gadchiroli and other rural districts in India with large tribal populations have been strongly associated with so-called ‘Naxalite’ anti-government groups. One issue of contention underscoring these groups is lack of land rights to tribal peoples [13]. Dissatisfaction with current government policies on such matters is expressed through attacks on government workers and infrastructure. Violence is a real and constant threat in these rural areas, and affected regions are unpopular posts for civil servants.

The remote locations of tribal villages in Gadchiroli district also pose a particular challenge to the NVBDCP malaria control strategies. For example, the NVBDCP standard for malaria testing states that slides should be examined by a laboratory technician within 24 hours of collection, and any positive results communicated to the patient within 48 hours so that appropriate anti-malarial medication can be initiated promptly [6]. The distance between villages poses a difficulty for maintaining this standard, as the health worker may not be able to complete weekly surveillance, or deliver the slides to the laboratory within 24 hours of collection.

#### Socio-Cultural factors

In tribal communities, healthcare seeking behavior is motivated by culturally specific beliefs about which practitioners to consult with regarding issues of health. For example, Vijayakumar et al. (2009) found that the tribal populations in Eastern India sought treatment for malaria symptoms from traditional healers first [14]. Also, a prior study in Gadchiroli district has suggested that local tribal peoples do not allow insecticide spraying in all rooms of the home, particularly where household altars to deities are located, thereby rendering the insecticide program ineffective [15]. Further, literacy rates are low among Indian tribal populations, and many tribal people in Gadchiroli do not speak the dominant state language of Marathi. Tribal children often leave school after the third or fourth year and “relapse into virtual illiteracy” [16]. Therefore educational materials presented by community health workers (who do not speak tribal languages) are not always comprehensible to tribal communities.

### Study design and sampling

This study sought to identify barriers to appropriate prevention and treatment for malaria infection among tribal communities in Gadchiroli district. Qualitative data were gathered from multiple key informants and groups to understand delivery, use and adherence with malaria prevention and treatment. Specifically, focus group discussions and interviews were performed with tribal people, traditional tribal healers (called *pujaris*), NVBDCP community health workers (CHWs), medical officers, and district health officials. Interviews and focus group discussions (FGDs) were organized around the following domains of inquiry: 1) the current understanding about malaria in tribal communities, and how that knowledge (or misconception) is transmitted 2) the treatments, if any, sought among tribal populations for fever within the initial 24 hours 3) the services or peoples consulted prior to allopathic practitioners by tribal people for fever, and in what ways these are perceived as effective or ineffective 4) the costs associated with treatment of fever/malarial infection among tribal communities; and 5) experiences and attitudes among tribal populations about the practices of IRS, use of ITNs, and use of anti-malarial medication.

We used random and purposive sampling for selecting villages and participants for this qualitative study. The aim was to maximize information-rich cases, as well as to represent the diversity of population and villages of the region. We selected five villages for FGDs (Table 1). Out of these, four villages were selected randomly, two each from two clusters of villages (with 23 and 19 villages each) in the field practice area of SEARCH using statistical software Stata (version 11, College Station, TX, USA). A fifth village (not within the SEARCH network) was located near one of the aforementioned villages, and was selected based on convenience. Male or female members present in the village were invited to participate in FGDs by authors RS and CG. The participation in the FGDs was open for all within the gender groups and the goal was to recruit 6–10 individuals for each FGDs. This was achieved in all but one FGD (male FGD in village D, see Table 2). Traditional healer, medical officer and district official interview participants were purposively selected. The participants for CHW FGDs were also purposively selected, and approached for enrollment after a monthly meeting at the primary health center. No *a priori* sample size was set for any participant group, and enrollment was continued until relative data saturation and information redundancy was achieved.

**Table 1:** Demographic data for villages included in the study.

Tribal villages	Estimated population	Distance from SEARCH HQ (in km)	Part of SEARCH program area?
Village A	75	42	Yes
Village B	150	32	No
Village C	200	10	Yes
Village D	200	13	Yes
Village E	165	49	Yes

**Table 2:** FGD respondent demographic information shown by village and gender.

Males (n = 28)	Village A	Village B	Village C	Village D	Village E
Age	35–60	21–75	22–60	18–37	19–50
FGD size	6	6	6	4	6
Number in household (persons)	6–12	2–13	4–8	5–7	3–8
Number in household <5 yrs old	0–2	0–5	0–1	0–1	0–2
Number in household pregnant	0	0–1	0	0	0
Highest formal education	None (5)8^th^ std (1)	None (3)2^nd^ std (1)5^th^ std (1)10^th^ std (1)	None (2)9^th^ std (2)12^th^ std (1)Masters (1)	8^th^ std (1)9^th^ std (1)10^th^ std (2)	None (2)2^nd^ std (1)4^th^ std (1)8^th^ std (2)
Households with electricity	2/6	1/6	5/6	1/4	3/6
Households with scooter	2/6	0/6	0/6	0/4	1/6
Annual income (in Rupees)	12,000–60,000	? – 9,000	10,000–25,000	20,000–60,000	? – 10,000

Females (n = 27)					
Age	25–55	18–50	27–65	19–60	-
FGD size	6	7	8	6	-
Number in household (persons)	5–12	1–9	4–7	1–8	-
Number in household <5 yrs old	0–2	0–1	0–1	0–1	-
Number in household pregnant	0	0	0	0	-
Highest education	None (3)5^th^ std (3)	None (5)5^th^ std (1)12^th^ std (1)	None (6)8^th^ std (1)10^th^ std (1)	None (3)9^th^ std (1)12^th^ std (2)	-
Households with electricity	2/6	1/7	7/8	2/6	-
Households with scooter	2/6	0/7	0/8	0/6	-
Annual income (in Rupees)	40,000–60,000	? – 10,000	? – 30,000	? – 18,000	-

Age is self-reported, in years. Number of participants in focus group, household size, number of household members under the age of 5 years, and number of pregnant members of household are shown. Education levels are shown as “standard”, which is equivalent to a primary or secondary school “grade” in the United States. The number of FGD participants reporting that highest level of education is shown in parenthesis. Number of households who report having electricity or owning a scooter is shown as fraction of total FGD participants from that village. Annual income is self-reported and often respondents were unsure of this figure (where ‘?’ is indicated).

### Data Collection

Data were collected in July 2012, during the beginning of the monsoon season and a peak period for malaria infection. Tribal villagers, CHWs, tribal *pujaris*, physicians practicing at district PHCs, and district NVBDCP officials were approached for participation in this study. Data were gathered through minimally-structured, face-to-face interviews and FGDs conducted in the village square, each lasting approximately one hour. Focus groups were held for tribal participants and CHWs, while all other groups were interviewed. Authors CG and RS conducted all FGDs and interviews. Both researchers were female.

Enrollment and FGDs for tribal focus groups took place directly in the villages, and were separated by gender to independently evaluate malaria knowledge among gender groups, and to prevent potential gender dynamics from preventing full participation from women. Inclusion criteria for participation in the tribal FGD were 1) 18 years of age or older, 2) willingness to give informed consent; and 3) self-identification as a member of a tribal group. All tribal FGD participants were members of the Gond tribe, the predominant tribe in this region. CHW FGDs were held at a PHC after a monthly meeting. *Pujaris*, physicians and district officials were approached in their places of practice for enrollment in this study. None of the CHWs, *pujaris*, physicians or district officials who were approached refused to take part in the study.

No prior personal relationships with any participants existed outside of the study. Prior to the interview or FGD, participants were told that the authors were affiliated with SEARCH, and interested in learning more about malaria. For participants who preferred to use the tribal language Gondi, real-time translation into Marathi was done by the SEARCH VHW during the focus groups, but this individual did not ask questions that were not posed by the interviewers.

### Ethics

This study was approved by the Institutional Research Board of the Brigham and Women's Hospital/Partners Healthcare and the Institutional Ethical Committee of SEARCH. All interview/FGD questions and informed consent were performed in a language participants could understand, typically Marathi or Gondi, through use of a native-speaking interpreter. District officials were interviewed in English, with use of a Marathi interpreter as needed. Verbal consent was used due to high rates of illiteracy among the study population and the Institutional Research Board of the Brigham and Women's Hospital/Partners Healthcare and the Institutional Ethical Committee of SEARCH approved this consent procedure. The verbal consent was witnessed by a literate person and a log of date, time and place of all FGDs was kept in the field notes for documentation purposes. No compensation was offered to study subjects for participation in this study. Interviews and FGDs were digitally recorded. The participants did not receive immediate feedback after the FGDs but the insights gained from this study will be shared with the tribal population through the tribal health program of SEARCH.

### Data Analysis

Data were analyzed using standard methods for qualitative studies [17], [18]. All FGD and interviews were transcribed from the digital recording into Marathi, then translated into English by one author, CG. The Marathi-to-English translations were verified by another author, YK, to ensure that translations were accurate and nuanced meanings from the original responses were not lost in translation.

Data validation was facilitated through investigator triangulation; open coding of all transcripts was performed independently by authors RS and CG to construct descriptive categories and extract content within the data that spoke to barriers to malaria control among tribal populations in Gadchiroli district. Categories and themes were not defined *a priori* to allow concepts to emerge within the narrative through the inductive approach, where “patterns, themes, and categories of analysis come from the data … rather than being imposed on them prior to data collection and analysis” [17]. Instead, categories and themes were iteratively developed, whereby reflecting upon and revisiting of the data by the researcher facilitates insight and helps develop meaning. Validity of these categories was reinforced via data triangulation, where independent respondents provided corroborating perspectives on the same topic. Data analysis was undertaken using the theoretical framework of *content analysis* to systemically organize data into a structured format [18].

No significant disparities in category construction between the two authors (CG and RS) were encountered upon comparison of independent open coding results. NVivo 10.0 software (QSR International Pty Ltd, Victoria, Australia) was used to assist with organization and management of qualitative data.

## Results

### Characteristics of the respondents

A total of 84 individuals took part in the study: 76 participated in focus groups, and eight provided individual interviews. Of focus group participants, 55 were village members, and 21 were community health workers. Nine tribal FGDs were held, separated by gender, and included four to eight participants each. In village E, only male participants were interviewed in order to reach equivalent numbers of male and female participants. Tribal FGD participants represented five villages in the district. Demographic information for tribal villages where focus groups were held is shown in Table 1. Villages included in this study ranged from ten to 49 kilometers away from the SEARCH headquarters with estimated populations between 75 to 200 persons.

Demographic information was collected for tribal FGD participants at the conclusion of the focus group, and is shown in Table 2. A total of 28 males and 27 females tribal participants were enrolled in the FGDs, with age ranging from 18 to 75 years among males, and 18 to 65 years among females. 12/28 males (43%) and 17/27 (63%) females reported no formal education. Electricity and ownership of mechanized transport were used as proxies for household wealth. Less than 50% of all participants had electricity (12/28 males and 12/27 female), and even fewer households owned scooters (3/28 males and 2/27 females). Individual interview study participants included four *pujaris*, two physicians stationed at PHCs, and two district NVBDCP officials.

### Barriers to malaria control

Our data reveal multiple barriers to malaria control, ranging from experiences at the village level extending to the functioning of NVBDCP initiatives within the district. These barriers fall into six broad categories 1) tribal knowledge about malaria, 2) reliance on traditional healers and informal providers for management of fevers, 3) surveillance, diagnosis, and treatment of malaria, 4) adherence to anti-malarial medications, 5) and malaria prevention with ITNs and IRS. We present a broader explanation of these barriers, showing that they are inextricable from socio-historical processes of cultural, economic and geographic marginalization (Table 3).

**Table 3:** Barriers to malaria control and the socio-cultural, economic and geographic factors contributing to these barriers.

	Barriers	Socio-cultural factors	Economic factors	Geographical factors
1.	Tribal knowledge about malaria is poor	1. Culturally inappropriate health education material2. CHWs do not understand or speak tribal language	1. Lack of access to education due to poverty predisposes to poor knowledge about malaria	1. Residence in remote locations prevents access to schools2. CHWs may not be able to reach villages on a regular basis and provide health education
2.	Heavy reliance on traditional healers and informal providers for evaluation of fevers	1. Belief in spiritual cause of physical symptoms2. Prior experience of rapid symptomatic relief with treatments from informal providers3. Prior experience of physicians not being present at the PHCs	1. Lack of mechanized transport due to poverty creates difficulties in accessing the PHCs or other formal providers	1. Difficulty accessing the PHCs due to remote area of residence
3.	Surveillance and diagnosis of malaria is inadequate	1. Delays in malaria diagnosis due to treatments from traditional healers first2. Vacant posts of NVBDCP officials due to insurgency factors in the district	1. Preferring locally available treatments to save costs of travel and lost wages	1. Health workers cannot make timely visits for surveillance especially during rainy seasons2. Delays in the diagnosis of malaria due to longer time needed to transport slides to a laboratory and get the results3. Stock-outs or other supply chain difficulties create medication shortages in rural areas
4.	Adherence to antimalarial medications is poor	1. Practice of stopping anti-malarials as soon as there is symptomatic relief2. Counseling by traditional healers not to take anti-malarials3. Oral medications are perceived as ineffective	1. Lack of education due to poverty predisposes to illiteracy, poor knowledge about malaria and poor adherence to medications	
5.	Malaria prevention with ITNs and IRS is inadequate	1. ITN use affected by cultural practices e.g. only males using ITNs due to their higher social status2. Use of ITNs for other purposes such as fishing3. Reluctance to IRS due to concern for contamination of belongings4. Not allowing IRS in rooms where household altars or deities are located	1. Poor purchasing capacity due to poverty decreases use of ITNs	1. Need to travel longer to purchase ITNs as they are not easily available in local markets

#### 1. Tribal knowledge about malaria is insufficient

We found that knowledge regarding malaria infection varies greatly among tribal villagers. There is no Gondi word for malaria. Instead tribal people utilize the English word ‘malaria’ or the Marathi term ‘hivtaap’. Table 4 shows the various physical symptoms that villagers and traditional healers associate with malaria infection. When asked about the vector, the overwhelming majority of respondents reply “mosquitoes”; however, there is diversity in explanation as to whether it is simply presence of mosquitoes or their bite specifically that transmits infection. Other villagers attribute malaria to “filth” or “germs that enter the body at night”. When asked about groups that should be considered high-risk for malaria infection, tribal participants replied, “everyone”, “children” and “women” (some replied pregnant women specifically). Villagers also consider the monsoon (July through September) and winter seasons as highest risk for contracting malaria, stating that cases are less common in the summer. Our data suggest that gender does not seem to affect the overall content of knowledge about malaria infection.

**Table 4:** Responses to the question “What are symptoms of malaria?” provided by tribal villagers and *Pujaris*, during FGD and interviews, respectively.

Tribal villager respondent	*Pujari* respondent
Fever with chills	Fever with chills
Chills	Giddiness
Headache	Loss of appetite
Pain in extremities/Difficulty walking	Constipation
Giddiness	Diarrhea
Diarrhea	Vomiting
Vomiting	Jaundice
Loss of consciousness/convulsions	
Yellow/dark urine	
Confusion/incoherence	
Dizziness	
Stomach ache	
Jaundice	

More frequent responses appear towards the top of the table.

The sources of tribal knowledge regarding malaria are heterogeneous. Participants report learning about malaria through CHW visits to the villages. Others learn from the medical practitioners at the PHCs, informal health care practitioners, and from the outreach program of SEARCH. As one female tribal FGD respondent said, “*Until we go to a doctor, we don't know anything. Doctor tests blood and tells us if we have malaria or not.*” The final source of education about malaria can be described as ‘experience’. A number of villagers reported that they knew about malaria having suffered it previously, or through discussions with friends, family or neighbors who have been infected:

*Interviewer: From where do you get this information (about malaria)?*

*Tribal Respondent: I know because I have suffered from malaria before. We discuss this among the villagers*. (Tribal FGD, male respondent)

Lived experience was a powerful source of knowledge about mosquitoes and malaria. For example, tribal participants demonstrated a clear understanding about the propensity for mosquitoes to breed in stagnant water. During a tribal focus group, one respondent stated:

*During winters, there are small insects on the surface of water bodies. We think these small insects further grow into mosquitoes. [Interviewer: How do you know this?] I saw larvae moving in water. The larva comes out of the cocoon and turns into a mosquito. Malaria is caused by mosquitoes, so the people say. That's how I know. I saw these bugs in water.* (Tribal FGD, male respondent).

Language barriers, geographic isolation and high rates of illiteracy contribute to current deficiencies in tribal knowledge about malaria. Currently, NVBDCP educational posters are in Marathi, with heavy reliance on text to communicate information to a largely illiterate population. Among our sample, 53% of participants reported no formal education with only one-third (18/55) completing education above the 5^th^ standard (see Table 2). Existing posters are inadequately distributed in rural areas. We only encountered one village with posters visible during our research period. There is currently no collaboration between NVBDCP and other governmental divisions to present this material in primary schools or other public venues.

In addition, current NVBDCP malaria educational programs are carried out by CHWs in the villages, where they often encounter a language barrier, as many CHWs do not speak Gondi, the local tribal language, and many villagers do not comprehend the dominant state language of Marathi.

#### 2. Tribal communities rely on traditional healers and informal providers for evaluation of fevers

Another significant barrier to early diagnosis of malaria is tribal reliance on traditional healers, or *pujaris*, for evaluation of malaria symptoms. These healers are present in most villages and are often consulted first on matters involving physical ailments. The role of *pujari* is passed down through male lineage in families, and ensconced in a traditional responsibility of service to tribal village inhabitants. Therefore no fees are charged for the services provided, and these individuals are highly respected within tribal communities. Rituals are performed along with administration of herbal remedies to treat any physical ailments. Common practice is to keep the patient in the *pujari*'s care for 2–3 days undergoing this treatment (though we heard of one case where the patient was kept with the *pujari* for two weeks). If symptoms persist or worsen, the patient is referred out of the village for further care and diagnosis, although in some cases, tribal villagers will visit another *pujari* in a neighboring village if symptoms do not improve.

Tribal communities believe that any physical illness can be caused by evil spirits, unfulfilled ancestral commitments, or *Atpata* (inauspicious arrangement of one's home). *Pujaris* can make interventions to resolve these conflicts and therefore “cure” the illness. When asked to describe his role in treating illness, a *pujari* replied,

*I check whether there is something wrong with one's Atpata. If it is so, I treat that. Otherwise I ask the patient to go to a clinic. Many times, their ancestors make some promises about sacrificing a hen or a pig, and if they fail to do so, any illness in the family is attributed to that.*

Although villagers and *pujaris* recognize they cannot treat malaria, villagers adamantly follow the practice of seeking counsel from the *pujari* first, and follow recommendation for referral to an allopath if rituals do not alleviate symptoms. Tribal participants endorsed primary concern that physical symptoms could be due to a spiritual illness that required treatment from *pujari*. The concern for malaria infection was secondary to this. A tribal FGD participant described a recent trip to the *pujari* for her daughter's malaria infection:

*Tribal Respondent: For two days he did all the rituals. He cannot do anything for malaria.*

*Interviewer: In spite of this why do you go to a pujari?*

*Tribal Respondent: Initially no one knows… neither I nor the pujari. Once we go to a clinic we come to know [whether we have malaria].* (Tribal FGD, female participant)

*People have faith in the pujari. For any disease they first go to the pujari. They go to a clinic if referred by a pujari. Some not only go to pujaris of their village but also from another village as well.* (CHW FGD participant).

These practices can be attributed to faith, adherence to tribal tradition, and the cultural authority that *pujaris* hold among tribal communities: “*You go to reputed doctors, spend lakhs (100,000s) of rupees, but don't get cured. For tribals like us, a person does not have recovery unless rituals are done*” (Tribal FGD, male respondent). Some CHWs reported experience with *pujaris* who counsel tribal villagers against taking medications and allowing blood tests by the CHWs.

Tribal participants also preferred so-called “Bengali doctors” who are informal healthcare providers. These are unlicensed practitioners (sometimes also called “private” doctors) who do not have any formal medical training, and do not follow standard practice for treating malaria. In some cases, these practitioners will perform blood testing and appropriately initiate malaria treatment. In other cases, they focus on symptom relief with saline infusions and intramuscular injections of non-steroidal anti-inflammatory medications.

These latter practices explain the heavy favor by tribal communities. In response to a question about why he refers his patients to “Bengali doctors” rather than PHCs, a *pujari* responded, “*In a private clinic, patient gets cured immediately. Not in the PHC. Private physicians give effective medicines*.” These practitioners are located closer to villages and often visit villages regularly, and therefore patients incur lower travelling costs. In fact, these practitioners will also canvass the villages for potential clients:

*Interviewer: How do you know that the treatment is available with the Bengali doctor [informal provider]?*

*Tribal respondent: He visits our village. His treatment cures our ailments so we know [it works].* (Tribal FGD, male respondent)

#### 3. Surveillance and treatment of malaria cases among tribal communities is inadequate

Our study showed a number of barriers to malaria diagnosis due to inadequate active surveillance of fever cases by CHWs at the village level. This process is complicated by the rural nature of the villages, lack of transportation facilities for CHWs, isolation of villages secondary to flooding of roads during the monsoon, and non-availability of RDKs in some areas. A CHW stated, “*We don't have transportation facilities. During rains, rivers are flooded; hence we often miss regular village visits.*”

In addition, the activity of Naxalite groups within the district often results in closure of roadways due to threats of violence. These groups also de-motivate NVBDCP staff from seeking employment in this region. A NVBDCP officer stated,

*With the vacancy positions in our district, with some insurgency factors, [malaria] surveillance is not weekly. In some areas it is fortnightly (every other week) … if we go with fortnightly visits, probably we will never be able to control [malaria].*

There is also delay to smear results, as large distances between villages, lack of transport, and vacancies in the district contribute to create a long delay between blood smear collection and results communicated back to the village level. A NVBDCP officer said,

*Examination [of the blood smear] is done within 14 days …. It has to be seen by a lab technician, even with RDKs. We are able to examine all the blood smears within 48 hours. From [CHW] to technician and then back [to the village], it takes 14 days … Distance is the problem. And transport is the problem.*

There are several additional barriers to adequate treatment of malaria under the NVBDCP program in this region. CHWs report shortage of anti-malarial medication supply to the PHCs for empiric treatment while final diagnosis is confirmed via blood smears. Also CHWs report being confused about the NVBDCP recommendations regarding treatment of malaria, particularly in light of the Artemisinin-based Combination Therapy (ACT) recall in the state of Maharashtra in early 2012, due to potential contamination of stock.

*The guidelines change very often. Initially we used to give chloroquine, and then came S-P [sulphamethoxazole/pyridoxine] and then ACT. Now they have banned ACT also. We are completely confused.* (CHW FGD respondent)

Understaffing in remote tribal region also leads to non-availability of medical officers at the PHCs. Tribal respondents recalled prior experiences with PHCs specifically when, after making a long journey to these centers, they found that no physicians were present to treat them.

*I was taken by a motorcycle to [a neighboring PHC]. The doctor was not there. Then we went to the PHC at [another nearby town]. That was also closed. Then we went to a Bengali doctor (informal provider) in [the same nearby town]. He gave two injections, one in each arm. He added the third injection to the saline [intravenous infusion].* (Tribal FGD, male respondent).

#### 4. Adherence to anti-malarial medications is poor among tribal communities

Our data indicate poor adherence to anti-malarial medications, as tribal respondents often discontinue medications if symptoms improve. In addition, there is reluctance among tribal communities to take oral anti-malarial medications, as they question the efficacy of oral medications in general. Tribal participants indicated that they discontinue anti-malarial medications due to perceived side effects, such as “giddiness” or because the tablets had a bad smell.

*Some [tribals] don't [complete the medication]. They stop the medicines as soon as the fever starts subsiding. They may take them for a day, and then they stop it.* (Tribal FGD, male respondent)

*Most of them [tribal patients] want [intravenous] injection or saline. If we refuse to give it to them, and we give them an oral tablet, they will take it and most of them don't use it.* (PHC medical officer)

Many CHWs attribute such non-adherence to illiteracy, and often will seek assistance from a literate household member or friend to ensure the medications are taken properly. A CHW stated,

*When people come to a clinic, they say ‘we first went to the pujari and therefore we had to come to a clinic (as they did not feel better after the rituals). When we got medicine from here, they were not effective and so we went to pujaris again.’ This is all because of superstitions and illiteracy.*

#### 5. Malaria prevention is inadequate: experiences with ITNs and IRS

ITNs were last distributed in Gadchiroli district by the NVBDCP in 2009. Our research found that ITN use is inconsistent and density is low among tribal communities. NVBDCP officials estimate that approximately 50% of tribal peoples actually use the nets appropriately. Others do not use them, or instead use these nets for fishing. Our respondents knew that ITNs are useful for reducing mosquito nuisance but did not demonstrate knowledge that this also reduced likelihood of malaria infection. Of tribal villagers who own ITNs, we noted heterogeneous use patterns. In some instances, ITNs are not used in the summer because they worsen the sensation of feeling hot. Others do not use them in the winter, because the villagers tend to sleep outside next to the fire during this season, and therefore do not use beds during that time of year. Within families, males tended to use the nets preferentially due to their higher status as head of the household. A tribal respondent indicated, “*Only males sleep under nets. Women don't. We use it more during winters and also in monsoon*” (Tribal FGD, male respondent).

Another aspect of the NVBDCP plan for malaria prevention is biannual spraying of Deltamethrin insecticide. Tribal respondents demonstrate skepticism about this practice, doubting its efficacy in controlling mosquitoes, and expressing concern about the insecticide's potential to contaminate or spoil possessions. Villagers also state that they do not allow spraying inside the kitchen or where household altars to deities are located for fear of contamination. Respondents also complained the spray had a “bad smell” and may give rise to physical side effects, such as a rash.

### Diagnosis and treatment for malaria outside the village are associated with higher costs

In Gadchiroli district, diagnosis and treatment of malaria occurs both inside and outside the village context. Tribal villagers report occasional diagnosis by CHWs within villages themselves, using RDK and blood smears. Villagers were also diagnosed outside the village at the PHCs, rural and district hospitals, by informal providers, or at the SEARCH clinic. Seeking medical care outside the village is associated with higher costs. Costs of travelling ranged from 40 to 150 rupees for a family. Lost wages ranged from 35 to 100 rupees daily, and patients reported missing anywhere from 1 to 14 days of work due to illness. This loss of income was not limited to the infected person, but extended to entire families, particularly if hospitalization for malaria treatment was required. Costs of medication ranged from 75 to 300 rupees. Where treatment costs were charged separately, this cost varied between 20 and 240 rupees. Respondents stated that infected children missed anywhere from one to two weeks of school due to malarial illness.

## Discussion

The problem of malaria control in the geographic and cultural contexts of Gadchiroli district is complex. Tribal communities in India are marginalized as the result of processes embedded in history, economics and society; interventions therefore must consider these realities and offer solutions accordingly. While the geospatial and cultural-historical processes that have shaped the lives of tribal communities in Gadchiroli district may be unique, the barriers we have identified here could potentially be extrapolated to similarly marginalized communities where malaria control remains a problem. The role of similar qualitative research in shaping the development of evidence-based prevention, management and control strategies has been demonstrated in similar cases worldwide [19], [20].

Our study identified several areas for improvement within the NVBDCP program. For the barriers to malaria control identified in this study, we discuss potential solutions.

### 1. Improving health education material

We found that the tribal knowledge about malaria is poor. The educational materials prepared by NVBDCP in the state rely heavily on Marathi text, which hints at ethnocentrism, and is not suited for the non-Marathi speaking and largely illiterate tribal population in this district. Dutta-Bergman (2005) has argued that health campaigns aimed toward marginalized groups often ignore the role of socio-cultural context and structural inequalities that shape or constrain individual behavior [21].

Creating new educational material in native language with pictograms, and increasing visibility of these posters or fliers among tribal communities and schools in tribal areas can help spread knowledge about malaria. These materials should attempt to convey the importance of a) prompt blood testing for malarial symptoms especially among high risk groups, b) medication compliance and c) use of preventive measures.

### 2. Working with traditional healers and informal providers

The impact of the cultural factors on malaria control in tribal communities is most evident in understanding the central role of the *pujari* in tribal communities. *Pujaris* are consulted first to exclude spiritual or ancestral causes of symptoms, and management at a clinic is initiated when the treatment from *pujari* has failed, anywhere from two days to two weeks later. This clearly creates an important barrier to timely diagnosis and treatment of malaria.

A potential avenue for further intervention will involve partnership with village *pujaris*. SEARCH has held health education seminars for *pujaris*, and our research suggests that these sessions are helping to some extent to facilitate knowledge and cooperation with CHWs when treating patients with malaria. The NVBDCP officials could also consider holding educational seminars for *pujaris* to educate them about a) the signs and symptoms of malaria and b) the importance of early diagnosis and treatment, particularly in pregnant women and children. Furthermore, when CHWs visit a village a crucial component of active surveillance would be to visit the *pujari* to inquire if any patient with fever is being under his care.

**Figure 2:**
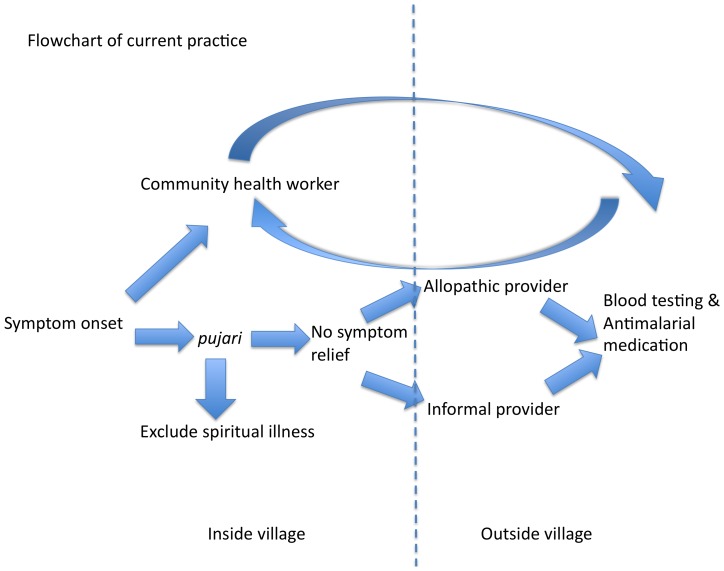
Flowchart demonstrating the current manner of diagnosis and treatment of malaria.

**Figure 3:**
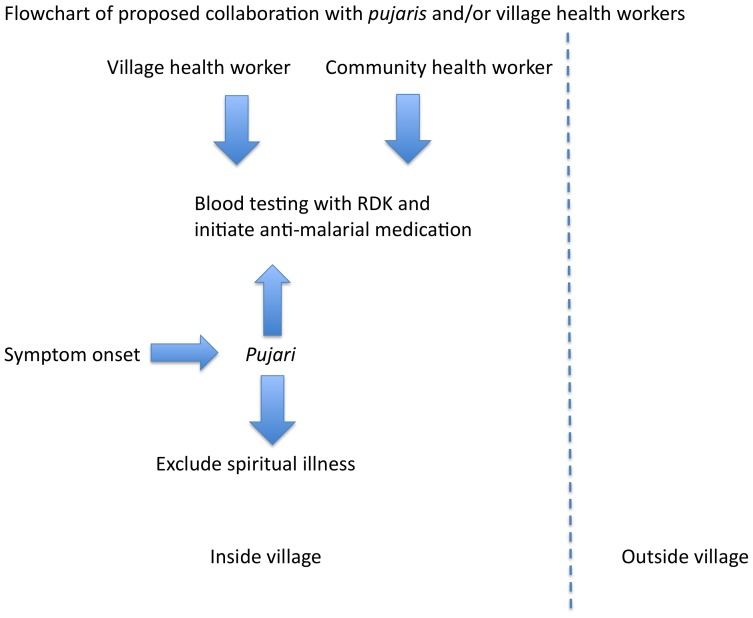
Flowchart demonstrating proposed collaboration with *pujaris*, for prompt malaria diagnosis beginning at the onset of symptoms in tribal village.

Given their vital role in influencing healthcare seeking *pujaris* should be considered a ‘bridge’ between tribal communities and allopathy, potentially to involve them as treatment partners for malaria cases. The goal is not to subvert the *pujari*'s culturally important role in excluding a spiritual case of the symptoms, but to have both arms of treatment underway concurrently within the village context (see Figures 2 and 3). That is to say, blood testing and anti-malarials can be initiated by a VHW who is a resident of the village (such as an Accredited Social Health Activist [ASHA]) on day 1 of fever symptoms while the *pujari* is conducting rituals to exclude the spiritual underpinnings of illness. If smear results are positive for *P. falciparum*, ACT could be initiated by the VHW, as the patient may still be under the care of the *pujari*. Another potential solution would be to train *pujaris* in using RDKs to diagnose malaria.

In many developing countries informal providers provide bulk of healthcare, especially for the poor [22]. The treatment provided by informal providers is often aimed at rapid symptomatic relief but may not adequately treat the underlying cause of illness. In order to ensure that patients with malaria are treated adequately with recommended medicines, NVBDCP could consider educational campaigns targeting these informal providers and training for informal providers in appropriate diagnosis and treatment of malaria.

### 3. Improving surveillance and diagnosis of malaria by strengthening the role of the VHW

In the remote, malaria endemic zones, diagnosis and treatment of malaria is often delayed (Figure 2). Also, during the rainy season when the incidence of malaria is high, many villages are cut off from the surrounding areas due to flooding and CHWs cannot access these villages. A health worker who is a resident of the village, such as an ASHA, can ensure ‘within village’ diagnosis and treatment of malaria, thereby reducing the delays in these processes (Figure 3). SEARCH has observed that RDK use by their tribal VHWs has led to increase in testing for malaria among fever patients. Patients feel satisfied that malaria can be diagnosed immediately and this is an empowering experience for both the patients and the VHWs. As such, increased funding and education for ASHAs or similar VHWs, either by the NVBDCP or through non-governmental organizations such as SEARCH, can improve the density of VHWs among tribal communities and strengthen their positions as central to the tribal healthcare-delivery process.

### 4. Ensuring adequate supply of rapid diagnostic kits and anti-malarial medications

We believe that rapid diagnosis of malaria and prompt initiation of treatment within the village context can help reduce malaria burden in remote areas in endemic zones. However, our data suggest that RDKs and anti-malarial medications are often not easily available. The NVBDCP program should ensure the availability of these materials in endemic areas, especially those areas with indigenous populations and remote locations, as in Gadchiroli district. Our data also show that deficiencies in the anti-malarial supply chain leads to decreased faith in the malaria control program by the CHWs. We believe that this negatively impacts the strength of the program and potentially reduces tribal confidence in CHWs for malaria symptoms.

### 5. Improving tribal adherence with anti-malarial medication

Adherence with anti-malarial medication is often very poor among the tribal population in the study area. This pattern of use results from equating presence of disease with the presence of symptoms, and patients often stop medications as soon as fever disappears or symptoms improve. This issue needs to be addressed while designing health education material and should be stressed while providing health education. Furthermore, the presence of a VHW can improve compliance by following up on patients who were begun on anti-malarial medications and ensuring that medications are taken properly.

### 6. Increasing the distribution of ITNs and promoting appropriate use

Distributing ITNs among tribal villages in Gadchiroli district may lower infection rates, as many participants did not have an ITN within the household. However, while ITN density is low in this region, it is clear that improving distribution will not directly lead to proper usage. Our study showed multiple barriers to proper use, including gender bias and seasonal sleeping patterns. Further, tribal participants did not directly associate ITN use with malaria prevention. Interestingly, Sood et. al. (2010) conducted surveys in rural (non-tribal) regions of Uttar Pradesh state of India that demonstrated >90% favorable attitudes towards ITN usage among these communities [23]. This contrasts with the trends of ITN use in tribal regions of Gadchiroli, highlighting the difference in behaviors between non-tribal and tribal communities, and underscoring the concept that tribal persons have patterns of ITN use that are distinct from rural (non-tribal) Indians. Therefore, along with ITN distribution, culturally appropriate health education should be provided towards improving ITN adherence.

### 7. Further research on tribal concepts of health and illness

We found that tribal respondents favored the treatments that alleviated symptoms most rapidly (such as intravenous saline or anti-inflammatory medicine), rather than treatments that *cured* the underlying pathogen. In some ways these practices may represent lack of knowledge about the potential morbidity and mortality of malarial infection. Alternatively, the choices to stop medication due to relatively mild side effects, or to seek care from practitioners that can provide quick symptom relief, provide insights towards the *moral* nature of tribal experiences [23]. Kleinman uses the terms ‘moral experience’ to describe experiences that illuminate what is most at stake for people, or make visible “values in ordinary living” [24], [25]. Our data hints at such deeper meaning behind such decision-making, and is a potential avenue for further data collection to better understand healthcare-seeking behavior and concepts of illness among tribal communities in Gadchiroli district.

### Study limitations

We recognize some limitations of our study. Our data and conclusions are based on participant responses, and therefore reflect what participants have told us, and cannot predict or verify behavior. As qualitative research aims to gather data from information-rich cases [17], rather than *representative* samples of the population as a whole, there is inherent bias in this methodology that excludes those less visible in the village, or unwilling to participate for whatever reason. Similarly, while we propose theoretical models for understanding the barriers to malaria control among these communities, we stop short of making broad conclusions about socio-cultural concepts of illness and health among these populations. Last, the responses of CHWs, *pujaris*, physicians, and district officials do not necessarily represent these groups as a whole. While we were able to achieve data saturation for most themes, we would likely have more diversity of responses with more participants. This is particularly true for groups for which a smaller number of participants were enrolled (*pujaris*, physicians and district officials).

## Conclusion

In conclusion, our qualitative study revealed that cultural, social and geographic, factors create barriers to malaria control among tribal communities in Gadchiroli district, who bear a disproportionate burden of malaria. Therefore it is crucial to understand these factors while developing effective malaria control strategies in such contexts. Our data indicate that management of malaria is likely to be successful when culturally appropriate education is used to improve understanding about malaria, traditional healers and informal providers are made partners in care of tribal patients to ensure early diagnosis and treatment, preventive strategies such as use of ITNs are promoted, and rapid diagnosis and treatment is ensured within the tribal villages themselves.

These findings have broad implications for the Indian NVBDCP program, as well as for international programs targeting marginalized populations living in endemic regions. While the historical and cultural processes at play may be unique to this region, the problem of addressing these barriers can be extrapolated to inform the improvement of malaria-control programs among similarly marginalized, resource-limited and underserved regions.
